# Availability of Ectomycorrhizal Fungi to Black Spruce above the Present Treeline in Eastern Labrador

**DOI:** 10.1371/journal.pone.0077527

**Published:** 2013-10-29

**Authors:** Laura Reithmeier, Gavin Kernaghan

**Affiliations:** 1 Biology Department, Dalhousie University, Halifax, Nova Scotia, Canada; 2 Biology Department, Mount St. Vincent University, Halifax, Nova Scotia, Canada; Institute of Botany, Czech Academy of Sciences, Czech Republic

## Abstract

Ectomycorrhizal fungi (ECMF) are an important biotic factor in the survival of conifer seedlings under stressful conditions and therefore have the potential to facilitate conifer establishment into alpine and tundra habitats. In order to assess patterns of ectomycorrhizal availability and community structure above treeline, we conducted soil bioassays in which *Picea mariana* (black spruce) seedlings were grown in field-collected soils under controlled conditions. Soils were collected from distinct alpine habitats, each dominated by a different ectomycorrhizal host shrub: *Betula glandulosa*, *Arctostaphylos alpina* or *Salix herbacaea*. Within each habitat, half of the soils collected contained roots of ectomycorrhizal shrubs (host**^+^**) and the other half were free of host plants (host^−^). Forest and glacial moraine soils were also included for comparison. Fungi forming ectomycorrhizae during the bioassays were identified by DNA sequencing. Our results indicate that ECMF capable of colonizing black spruce are widespread above the current tree line in Eastern Labrador and that the level of available inoculum has a significant influence on the growth of seedlings under controlled conditions. Many of the host^−^ soils possessed appreciable levels of ectomycorrhizal inoculum, likely in the form of spore banks. Inoculum levels in these soils may be influenced by spore production from neighboring soils where ectomycorrhizal shrubs are present. Under predicted temperature increases, ectomycorrhizal inoculum in soils with host shrubs as well as in nearby soils without host shrubs have the potential to facilitate conifer establishment above the present tree line.

## Introduction

The elevational and latitudinal limits of the boreal forest (treeline) are expected to expand with increasing temperature and longer growing seasons, resulting in the encroachment of seedlings into habitats currently supporting tundra vegetation [1], [2]. However, seedling establishment is also facilitated by a number of direct and indirect biotic factors [3]–[5], including the availability of ectomycorrhizal fungi (ECMF) [6], which colonize the roots of woody plants in a mutually beneficial symbiosis.

ECMF colonization improves the efficiency of water and mineral nutrient acquisition [7], [8] and can also provide access to organic nutrient sources that may otherwise be unavailable to the plant [9]–[11]. ECMF are therefore especially important for seedling establishment in arctic and alpine soils, where cold temperatures can make water inaccessible through frost drought and slow microbial mineralization rates result in nutrients being bound to accumulated organic matter [12]–[15]).

An important inoculum source for newly established seedlings is the dense network of fungal mycelia extending from and connecting to the roots of previously established ectomycorrhizal plants. These “common mycorrhizal networks” facilitate resource sharing between established plants and can also be important for seedling recruitment [16]. Seedlings establishing in close proximity to mature ectomycorrhizal host trees often exhibit better growth and survival, as well as greater ECMF colonization and species richness than seedlings establishing at greater distances from ECM trees [17]–[19].

Although there are few conifers to support common ectomycorrhizal networks above treeline, there are several woody angiosperm shrubs, including members of the Betulaceae, Ericaceae, Rosaceae and Salicaceae, which support ECMF that may be available to establishing conifer seedlings [20]–[25]). For example, the majority of ECMF species on *Arctostaphylos uva-ursi* (Bearberry) were also found on nearby *Pseudotsuga menziesii* (Douglas fir) seedlings [26] and the dominant ECMF colonizing alpine *Salix* and *Dryas* were found to be a subset of those colonizing treeline conifers [27]. Also, regenerating *Betula* and *Larix* saplings coincided with patches of *Salix reinii* shrubs on Mount Fuji, and the ECMF species colonizing the saplings were very similar to those of *S. reinii* [28]. Furthermore, when *Betula* and *Larix* seedlings were planted into habitats with or without *Salix*, only the seedlings planted into *Salix* patches exhibited extensive ECMF colonization [29].

Spore dispersal is also an important source of fungal inoculum for establishing seedlings [19], [30]. Dispersal may be by wind [31]), soil fauna [32], or mammals that consume the sporocarps of mycorrhizal fungi [33], [34]. Spore dispersal results in “spore banks” within soils which can accumulate and persist for several years [35]–[37].

The objective of the present study was to assess the potential availability of ECMF to *Picea mariana* (black spruce) seedlings above the present tree line, with a particular focus on the influence of ectomycorrhizal shrubs. We grew black spruce seedlings in soils collected from plant communities at different elevations in the Mealy Mountains of Labrador. Soils were chosen on which *Arctostaphylos alpina, Betula glandulosa,* or *Salix herbacea* had either established, or had not established. Forest soils and glacial moraine were also used for comparison. Due to very high mortality of conifer seedlings previously out-planted into these habitats, we chose a soil bioassay approach, in which black spruce seedlings were grown as ECMF “bait” in field-collected soils under controlled conditions. This approach allowed us to characterize the ECMF with the potential to colonize spruce in alpine soils, and also determine the influence of these fungi on the growth of seedlings. Although ECMF colonization in soil bioassays tends to be biased towards early successional fungi, these fungi are likely to be particularly important in the facilitation of conifer migration, as they are the first to colonize establishing seedlings.

## Materials and Methods

### Research Area

The Mealy Mountains are situated southeast of Lake Melville, Labrador, Canada and are expected to be particularly sensitive to climate change as they represent an isolated subarctic highland region at a relatively low latitude [38]. The study area lies within the Mealy Mountains/Akamiuapishk^u^ National Park Reserve (N 53°36.6′ W 58°49.0′), but at the time of sampling, the area was still classified as provincial land. No specific permission was required and no protected species were endangered. The area consists of a broad valley and a 1057 m a.s.l. peak, with vegetation grading from boreal forest to tundra.

The forest is dominated by *Picea mariana* but includes *Picea glauca*, *Larix laricina* and *Abies balsamea.* The forest-tundra ecotone is dominated by *Betula glandulosa* and several ericaceous species. The tundra is characterized by a layer of discontinuous permafrost [38] and is dominated by low-lying evergreen shrubs (e.g. *Salix*) [39]. Along the elevational gradient from the valley bottom to the mountain peak, the dominant ectomycorrhizal host plants grade from the forest trees through *Betula glandulosa* just above treeline to *Arctostaphylos alpina* at intermediate elevations and finally to S*alix herbacea* which dominates the tundra habitat. Average annual temperatures between 2001 and 2004 (recorded by automatic climate stations located at 570 m and 1000 m a.s.l.) were −1.8°C and −4.5°C, respectively [38].

### Soil Sampling and Analysis

Soil sampling was conducted within each of the three distinct alpine vegetation zones, or “habitats” (dominated by *Betula*, *Arctostaphylos* and *Salix*), located at least one km apart along an elevational gradient beginning at treeline. Within each habitat, five 10×10 m collecting sites were established, approximately 100 m apart. All sites were established within boulder fields and all soil samples were collected either on boulder tops or in crevasses between boulders, ensuring that they were physically isolated and did not contain roots from any surrounding ectomycorrhizal plants. Soil samples were also collected from five sites located in the sub-alpine black spruce forest, and one site located on a glacial moraine, at least 30 m from the nearest vegetation. Soil temperature and moisture were measured in the rooting zone with a Hanna Instruments surface probe (Rhode Island, USA) and a DeltaT HH2 moisture meter (Cambridge, UK), respectively.

Six soil samples (approx. 1 L each) were collected from within each site; three from random microsites supporting ectomycorrhizal shrubs (host**^+^** soils) and three from random microsites lacking ectomycorrhizal shrubs (host^−^ soils), although only host^+^ soils were available in the forest and only host^−^ soils were available on the glacial moraine. A total of 109 soil samples were collected; 30 in each of the *Salix*, *Arctostaphylos*, and *Betula* habitats, 15 in the forest and four in the glacial moraine. Tools used to collect soils were sterilized with a dilute bleach solution and rinsed with water between samples to avoid cross-contamination. Soil samples were stored in sealed plastic bags and kept cool until being shipped to the laboratory, where they were kept at 4°C for one month prior to analysis and planting. A portion of each soil sample was sent to the Nova Scotia Department of Agriculture for standard nutrient analysis including pH, cation exchange capacity and organic matter, total nitrogen, nitrate, phosphorus, potassium, calcium, magnesium, sodium, sulfur, aluminum, iron, manganese, copper, zinc, and boron contents.

### Mycorrhizal Bioassays

In order to assess ECMF inocula (percent colonization, fungal species richness, diversity, and species composition), mycorrhizal bioassays were conducted by growing black spruce seedlings as bait plants in each soil sample under controlled conditions. The soil remaining from each sample after nutrient analysis was used to fill four 2.5 inch pots (100 cm^3^ each) and planted with locally collected black spruce seeds from the Goose Bay Tree Nursery (Province of Newfoundland and Labrador Dept. of Natural Resources). Although the ectomycorrhizal mycelium would have been disrupted during sampling, many ECMF species are able to readily colonize plant roots from hyphal fragments and ectomycorrhizal root tips [40], [41], the latter potentially persisting as an inoculum source for several months after being disconnected from the original plant [42], [43].

Spruce seeds were surface sterilized in 15% hydrogen peroxide for one hour, rinsed with sterile water and germinated on sterile moist filter paper under fluorescent lights. Germinated seeds were then planted in sterile vermiculite within clean Conviron ATC26 growth chambers and grown at 20°C, 80% humidity and fluorescent light at 200 µmol for 18 hours per day. After one month, 85% of the seedlings were randomly transferred to the pots containing the field soils, which were placed in trays covered with clear domed lids for further growth and ECMF colonization under the same chamber conditions. The remaining 15% of the seedlings were left growing in the sterile vermiculite to serve as controls for ECMF contamination and growth chamber performance. Seedlings were given sterile distilled water as needed over the course of the bioassay.

### Analysis of Seedlings and Mycorrhizae

After 22 weeks of growth, seedlings were harvested, cleaned, and scanned on a HP Scanjet 4370 image scanner. Total root lengths were measured from the scanned images using WinRhizo version 2009b software (Regent Instruments Inc., Québec, QC). The shoot and root systems were then separated at the root collar and the shoots were oven-dried and weighed. Root mass data was not collected, as drying the root system was not compatible with the mycorrhizal assessment. Root systems from each bioassay pot were cut into 0.5 to 1 cm lengths and randomly distributed within a water-filled tray marked with a grid of 2×2 cm squares. Root tips within randomly selected squares were collected until 100 tips per pot were obtained. Dead and dying root tips (dark, wrinkled and brittle with loosely adhering epidermal cells) were counted and removed and the remaining tips analyzed microscopically in order to determine the percent of active root tips colonized. Active ectomycorrhizal root tips were then categorized into morphological groups (morphotypes) [44]–[47].

For commonly occurring morphotypes (those found in over 1/3 of the soil samples from any given habitat), one sample representing each morphotype from each of the five plots was selected for DNA sequencing. For less common morphotypes, a single sample was randomly selected for sequencing. For each selected morphotype, DNA was extracted from six to ten root tips using either the DNeasy plant mini kit (Qiagen Ltd., Toronto, ON) or the Wizard SV genomic DNA purification system (Promega Corp., Madison, WI). PCR amplification utilized the fungal specific primer sets ITS1-F/ITS4 [48] and NSA3/NLC2 [49], which target the internally transcribed spacer (ITS) region of the fungal rDNA without amplifying host plant DNA.

PCR amplification involved 50 µL reactions containing 25 µL GoTaq master mix (Promega), 15 µL of undiluted DNA extract, and 2.5 µmol of each primer. Temperature cycling was done with a Veriti 96 Well Thermal Cycler (Applied Biosystems, Carlsbad, CA). Cycling parameters were as follows when using the ITS1-F and ITS4 primers: an initial denaturation step of 95°C for 3 min., followed by 35 cycles of denaturation at 95°C for 1 min., annealing at 53°C for 1 min., and extension at 72°C for 2 min. and a final elongation at 72°C for 7 min. For the NSA3 and NLC2 primer set, cycling parameters were as above except that the number of cycles was reduced to 30 and the annealing temperature was raised to 67°C [49]). The sizes and concentrations of the resulting PCR product were determined on 2% ethidium bromide stained agarose gels.

Sequencing of PCR products was carried out at the McGill University and Génome Québec Innovation Centre with an ABI PRISM 3730XL DNA Analyzer system with ITS1 and ITS4 primers. Forward and reverse sequences were assembled into contigs using Sequencher version 4.9 software (Gene Codes Corp., Ann Arbor, MI). Sequences were aligned in MUSCLE Ver. 3.8.31 [50] and 97% similarity sequence groups identified using Bioedit Ver. 7.0.9.0 [51]. Fungal species were identified by comparison to reference sequences available in the UNITE [52] and GenBank public sequence databases using nucleotide BLAST (blastn) [53]. Although a similarity between the contig and reference sequence of ≥97% was considered a match at the species level [54], some morphotypes appeared to represent more than one closely related species but were pooled under a single morphotype designation as they were not separable on the basis of morphology. All sequences were deposited in GenBank (KC702613-KC702666).

### Statistical Analyses

Morphotype diversity indices (Fisher’s alpha) were calculated for each individual seedling grown in each soil sample using PAST 2.09 [55]. In order to graphically analyze the distributions of ECMF morphotypes in relation to habitat and host plant presence/absence, detrended correspondence analysis (DCA) was performed using CANOCO 4.53 (Microcomputer Power, NY). Confidence ellipses were calculated for the site scores for each of the four host^+^ soils and for all host^−^ soils combined using Systat 13 (SYSTAT Software, Inc., Chicago, IL). To assess differences in ECMF communities between seedlings grown in host**^+^** and host^−^ soils, two-way permutational multivariate analyses of variance [56] were conducted for each habitat type using PERMANOVA software (Available: http://www.stat.auckland.ac.nz/~mja. Accessed 2013 Sept 18) using Bray-Curtis distances and 4,999 permutations. Plots were used as one factor and host presence/absence as the other. Indicator species values [57] were also calculated for fungal species within each habitat using PC-ORD 4.26.

Data on soil factors (e.g. nitrate and phosphorus), ECMF factors (colonization, richness and diversity) and seedling factors (shoot mass and root length) were averaged across the four replicate bioassay pots from each soil sample. A series of two-way ANOVAs, followed by Tukey’s HSD post-hoc multiple comparisons tests, were used to compare the levels of these factors among sites and between host^+^ and host^−^ soils within each habitat. Among habitat differences were not analyzed by ANOVA, as habitats could not be effectively replicated.

In order to investigate the relative importance of ECMF on bioassay seedling growth, we conducted simple linear regressions of ectomycorrhizal factors against seedling shoot mass and root length. Separate regressions were conducted for all soils from each of the three above-treeline habitats individually, as well as for all host^+^ soils, all host^−^ soils, and all soil types together. Slopes of the resulting regression lines were compared by ANCOVA in Past 2.09.

In an effort to account for the variation not explained by the simple linear regressions, multiple linear regressions were performed (using the same habitat combinations as above) in which the dependent variable was either shoot mass or root length and the initial set of independent variables included: percent ECMF colonization, ECMF richness, ECMF diversity per seedling (Fisher’s alpha), soil pH, soil cation exchange capacity, soil organic matter content, levels of macronutrients (total nitrogen, nitrate, phosphorus and potassium) and a range of micronutrients. Multiple regressions were carried out for the same groupings of soil samples as used for the simple regressions. Initial Pearson correlations identified independent variables with significant simple linear relationships with the dependent variables. After assessing for collinearity, these independent variables were used to construct a series of potential regression models. Regressions and subsequent model selection on the basis of Akaike’s information criterion (AIC) were conducted in Systat 13. Values for shoot mass and percent ECMF colonization were log and arcsine transformed, respectively.

## Results

### Soil Factors

During the sampling period, *Arctostaphylos* soils were on average 6°C warmer and 43% dryer than *Salix* and *Betula* soils. Within habitats, soil temperatures were similar between host^+^ and host^−^ soils, except for *Salix*, in which host^−^ soils were 2.5°C warmer than host^+^ soils. Soil moisture was similar between host^+^ and host^−^ soils in the *Betula* habitat, but approximately 12% higher in host^−^ *Salix* soils and 50% higher in host^−^ *Arctostaphylos* soils. Average nitrate levels were highest in the *Salix* soils, lowest in the forest soil and similar between *Arctostaphylo*s and *Betula* soils. Within habitats, nitrate levels were significantly higher for host^−^ soils than host^+^ soils in both the *Arctostaphylos* and *Salix* habitats. Average soil phosphorus levels were highest in the *Salix* habitat, followed by *Betula,* then forest and *Arctostapylos*. Within habitats, soil phosphorus was significantly higher in *Betula* host^+^ soils than in *Betula* host^−^ soils (Table 1, Table S1).

**Table 1:** Average values for selected soil nutrients, ECMF factors and bioassay seedling growth from host^+^ and host^−^ soils within each habitat.

	Forest	Betula		Arctostaphylos		Salix	
	Host^+^	Host^+^	Host^−^	Host^+^	Host^−^	Host^+^	Host^−^
Nitrate(ppm)	0.72	2.66	5.71	**1.40**	**7.13**	**2.14**	**19.55**
	(0.20)	(1.15)	(1.85)	**(0.41)**	**(1.16)**	**(0.57)**	**(3.76)**
Phosphorus(ppm)	43.13	**72.06**	**46.46**	43.6	33.1	68.96	56.1
	(4.46)	**(11.25)**	**(5.60)**	(5.57)	(3.82)	(6.01)	(6.05)
% ECMcolonization	72.92	**71.82**	**49.26**	73.39	68.51	95.07	85.87
	(4.77)	**(5.76)**	**(8.91)**	(5.03)	(7.31)	(1.60)	(5.35)
ECMdiversity^a^	0.39	**0.30**	**0.19**	0.29	0.22	**0.34**	**0.20**
	(0.03)	**(0.04)**	**(0.02)**	(0.03)	(0.02)	**(0.01)**	**(0.01)**
Rootlength (mm)	84.61	69.19	71.80	**53.27**	**70.49**	57.71	73.62
	(7.49)	(6.07)	(5.39)	**(4.14)**	**(4.09)**	(5.76)	(4.83)
Shootmass (mg)	48.76	**76.98**	**40.69**	19.19	29.82	72.2	86.06
	(9.07)	**(14.6)**	**(9.69)**	(2.37)	(5.53)	(7.84)	(12.22)

Standard errors are in parentheses.

Significantly different within habitat values are in bold (α = .05).

a Fisher’s alpha.

### Availability of ECMF

Overall colonization levels of bioassay seedlings were relatively high when grown in both host^+^ and host^−^ soils, with the exception of those grown in glacial moraine soil, which remained uncolonized throughout the experiment. The moraine soil seedlings also grew very poorly and were not included in further analyses. Percent colonization was generally higher on seedlings grown in host^+^ soil than host^−^ soil but the difference was significant only for *Betula* soils (Table 1). Colonization rates were also positively correlated between host^+^ and host^−^ soils within habitats; i.e. colonization in both host^+^ and host^−^ *Betula* soils were lowest and both host^+^ and host^−^ *Salix* soils were highest (Table 1). Control seedlings did not become colonized by ECMF.

A total of 15 ECMF morphotypes were found on bioassay seedlings grown in the four soil types (*Arctostaphylos*, *Betula*, *Salix* and forest)(Table 2). Fourteen were identified by DNA sequencing, and one (*Inocybe*-like), for which sequencing was repeatedly unsuccessful, was identified on the basis of comparison with published morphological descriptions. Sequences from one particularly common morphotype consistently matched either *Laccaria* or *Thelephora*. As the mycorrhizae formed by these two fungi are difficult to distinguish before their mantles have fully developed [44], they were pooled for all analyses. Although this results in a small systematic underestimate of ECMF diversity, it was unavoidable given that our method relied on initial morphological characterization prior to sequencing.

**Table 2:** Examples of matches between sequences obtained from bioassay seedling ECM and public sequence databases (one example of each morphotype from each soil type).

Morphotype name	Sample code^a^	Closest data base match^b^	Similarity
*Cenococcum* ^1^	B1pos1.2_5	*Cenococcum geophilum*; AY394919	533/535 (99.6%)
*Elaphomyces* ^2^	F4pos1.1_43	*Elaphomyces muricatus*; UDB000092	666/678 (98.2%)
*Elaphomyces* ^2^	B4pos3.4_35	*Elaphomyces muricatus*; UDB000092	614/626 (98%)
*Hydnotrya* ^3^	F1pos3.2_44	*Hydnotrya cubispora*; EU784273.1	658/674 (97.6%)
*Inocybe* ^4^	A1neg3.2_13b	*Inocybe lacera*; AM882816	538/576 (93.4%)
*Laccaria*/*Thelephora* ^5^	A5neg2.2_13a	*Laccaria laccata*; UDB000106	691/691 (100%)
*Laccaria*/*Thelephora* ^5^	F5pos2.3_45	*Laccaria laccata*; FJ845416	688/690 (99.7%)
*Laccaria*/*Thelephora* ^5^	S4pos2.1_11	*Laccaria laccata*; UDB000106	684/684 (100%)
*Laccaria*/*Thelephora* ^6^	B2neg2.2_30	*Thelephora terrestris*; HM189964	662/663 (99.8%)
*Laccaria*/*Thelephora* ^6^	B3pos2.3_30	*Thelephora terrestris*; HM189964	639/642 (99.5%)
*Laccaria*/*Thelephora* ^6^	S2neg1.1_3	*Thelephora terrestris*; JQ711777	626/645 (97%)
*Lactarius* ^7^	B1pos1.2_29b	*Lactarius tabidus*; HM189833	683/731(93.4%)
*Lactarius* ^8^	F2pos2.3_47	*Lactarius rubrocinctus*; JF908273	652/672(97%)
*Meliniomyces* ^9^	F5pos2.4_52	*Meliniomyces bicolor*; HQ157926.1	518/534 (97%)
*Peziza* ^10^	S5pos3.1_12	*Peziza badia*; DQ384574.1	591/601 (98.3%)
*Pseudotomentella* ^11^	F5pos1.1_51	*Pseudotomentella tristis*; UDB000029	662/665 (99.5%)
*Sebacina* ^12^	A2pos3.2_23	*Sebacina* sp.*;* AF465191.1	582/626 (92.9%)
*Sebacina* ^12^	F1pos1.1_39	*Sebacina* sp.; AF465191.1	582/632 (92%)
*Sebacina* ^13^	B2pos3.2_37	*Sebacina* aff. *epigaea*; MW 526; AF490393.1	598/615 (97.2%)
*Sebacina* ^13^	S2neg2.2_9	*Sebacina* aff. *epigaea*; MW 526; AF490393.1	531/547 (97%)
*Tomentella* ^14^	A5pos1.3_2	*Tomentella stuposa*; UDB001660	631/635 (99.3%)
*Tomentella* ^15^	A4neg3.2_17	*Tomentella* sp.; HM189968	655/671 (97.6%)
*Tomentella* ^16^	B2pos3.2_2	*Tomentella ramosissima;* U83480	635/646 (98.2%)
*Tomentellopsis* ^17^	A3pos1.4_16	*Tomentellopsis submollis;* UDB016634	662/688 (96.2%)
*Trichophaea* ^18^	F4pos2.4_41	*Trichophaea* cf *hybrida* KH0439; DQ200834	548/575 (95.3%)
*Tylospora* ^19^	A4pos1.2_24	*Tylospora fibrillosa*; AF052562	589/590 (99.8%)
*Tylospora* ^19^	F3pos3.4_46a	*Tylospora fibrillosa*; AF052562	595/596 (99.8%)

a First letter, habitat; first number, plot; neg, host^−^; pos, host^+^.

b Accession numbers beginning with UD refer to the Unite Data Base, all others are from GenBank.

Superscripts after morphotype names indicate 97% sequence similarity groups.

**Figure 1:**
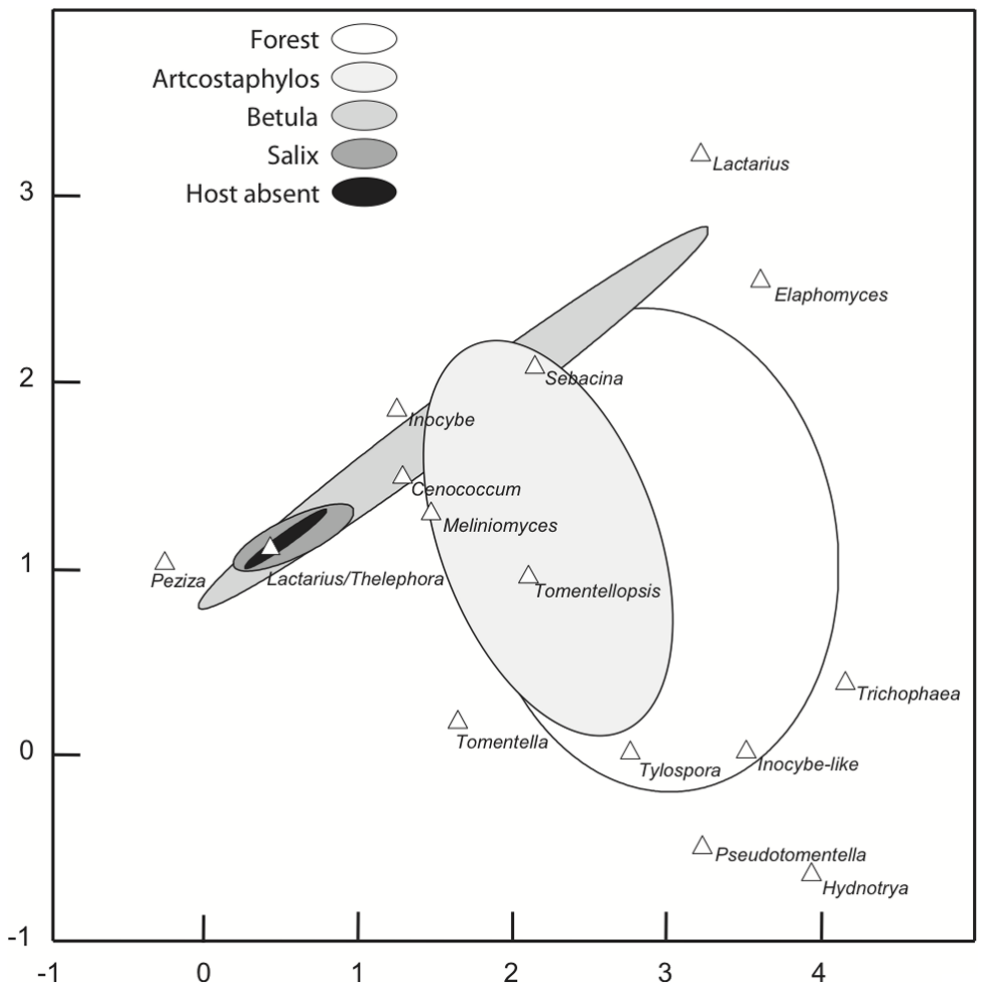
Detrended correspondence analysis (DCA) indicating differences in ECMF composition across different soil types. The four host^+^ soils are included separately and data from all host^−^ soils is combined. Confidence ellipses encompass the site scores (seedlings) grown in each soil type.

In the ordination (DCA) of soil samples and ECMF morphotypes (Fig. 1), the first and second axes explain a total of 26.5% of the variation in the data (17.4% and 9.1% respectively; λ_1_ = 0.834, λ_2_ = 0.435, total inertia = 4.792). The ECMF communities of *Arctostaphylos* host^+^ soils, *Betula* host^+^ soils and forest soils were relatively distinct, while the *Salix* host^+^ soils and all the host^−^ soils were similar to each other (Fig. 1). In fact, regardless of habitat, the ECMF communities from all host^−^ soils were similar to one another and generally represented a sub-set of neighboring host^+^ soil species (Table S2).

Multivariate analyses of variance indicates that within each habitat, ECMF communities are similar among plots, but differ significantly between host^+^ and host^−^ soils (Table 3). In the case of *Salix*, however, the significant plot x host presence/absence interaction indicates that *Salix* host^+^ and *Salix* host^−^ ECM communities are not significantly different on all plots. Indicator species analysis also shows that while *Cenococcum, Sebacina* and *Tylospora* are characteristic of host^+^ soils in certain habitats, the *Laccaria/Thelephora* morphotype is highly characteristic of all host^−^ soils (Table 4).

**Table 3:** Results of multivariate analyses of variance comparing ECM communities among plots and between host present and host absent soils (Host^±^) within each alpine habitat.

Habitat	Plot	Host^±^	Plot x Host^±^
*Salix*	0.764	**0.014**	**0.0436**
*Arctostaphylos*	0.643	**0.005**	0.511
*Betula*	0.765	**0.017**	0.211

Bold values are significant (α<.05).

**Table 4:** Results of indicator species analyses for morphotypes with significant indicator values in one or more habitats.

Morphotype	*Salix*	*Arctostaphylos*	*Betula*
*Cenococcum*	+85.1***	+27	+31.2
*Laccaria/Thelephora*	−56.0***	−92.0***	−66.2**
*Sebacina*	+12.1	+77.8***	+56.5**
*Tylospora*	np	+80.0***	np

** p<0.01;

*** p<0.001.

+ host present soils;

− ,host absent soils.

np, not present.

ECMF richness decreased with distance from the forest, with seedlings grown in forest soils supporting 11 morphotypes, seedlings grown in *Betula* and *Arctostaphylos* soils supporting seven morphotypes and *Salix* grown seedlings supporting only five. Also, *Betula* shared three morphotypes exclusively with the forest, while *Arctostaphylos* and forest soils shared only one. No morphotypes were shared exclusively between the forest and *Salix* (Table S2). The average ECM morphotype diversity per seedling was relatively high for forest and *Salix* soils and significantly higher in host^+^ than in host^−^ soils for both the *Betula* and *Salix* habitats (Table 1). However, as habitats were not replicated, we cannot be certain that the observed differences in ECMF communities among different elevations are driven solely by vegetation.

### Influence of ECMF on Seedling Growth

Within habitats, shoot mass was not significantly different between seedlings grown in host^+^ and host^−^ soils from the *Arctostaphylos* and *Salix* habitats, but was significantly greater in host^+^ soils than host^−^ soils for the *Betula* habitat (Table 1).

**Figure 2:**
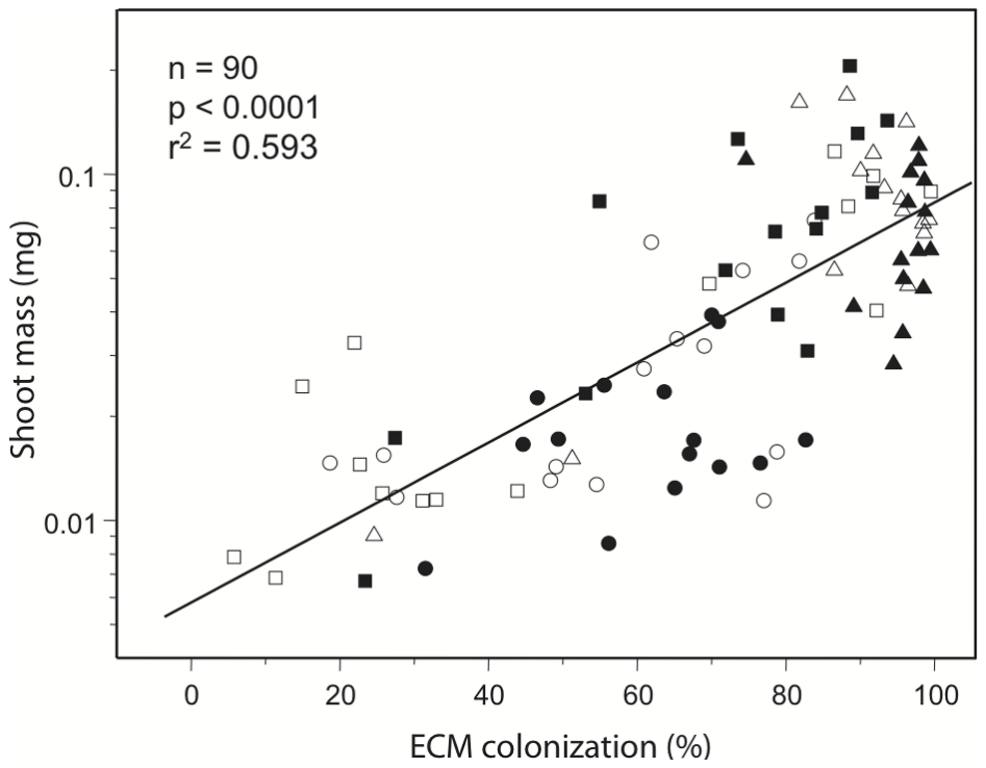
Linear regression of shoot mass of bioassay seedlings against ECM colonization level. *Arctostaphylos* soils; circles, *Betula* soils; squares and *Salix* soils; triangles. Closed symbols; host^+^ soils, open symbols; host^−^ soils.

Simple linear regressions of ECM factors against bioassay seedling shoot mass for five different groupings of soil samples indicated that although ECMF richness and diversity were not correlated with seedling biomass, percent ECM colonization and seedling shoot biomass were significantly correlated for all combinations of soil samples (Table 5). The simple linear regression using all alpine soil samples (n = 90) explained 58.5% of the variation in the shoot biomass data (Fig. 2), the regression using only host^−^ soils explained 68% of the variation and the regression using only host^+^ soils explained 48.2%. In regressions using only soils from the individual habitats, ECM colonization explained 74.3% of the variation in shoot mass grown in *Betula* soils, 22.7% for *Arctostaphylos* soils and 38.9% for *Salix* soils.

**Table 5:** Results of simple linear regressions of bioassay seedling shoot mass (dependent) against ECMF factors (independent) for different groupings of soil samples.

Origin of bioassay soil	n^a^	ECM colonization			ECM richness		ECM diversity	
		r^2^	p	slope^b^	r^2^	p	r^2^	p
All habitats (host^+^ and host^−^)	90	0.585	**<0.000**	0.620	0.008	0.401	0.006	0.475
All habitats (host^+^ only)	45	0.482	**<0.000**	0.485	0.010	0.506	0.071	0.077
All habitats (host^−^ only)	45	0.680	**<0.000**	0.742	0.000	0.929	0.019	0.369
*Betula* habitat	45	0.743	**<0.000**	0.688	0.006	0.207	0.038	0.300
*Arctostaphylos* habitat	45	0.227	**0.008**	0.342	0.009	0.625	0.010	0.608
*Salix* habitat	45	0.389	**0.000**	0.456	0.035	0.319	0.036	0.315

Forest and moraine soils are not included.

Statistically significant p values are in bold (α = 0.05).

Data on seedling mass and ECM colonization were log and arcsine transformed, respectively.

a Number of soil samples included in each grouping.

b Slopes are included for significant regressions only.

Simple linear regressions also illustrate the differences in the relationship between percent ECM colonization and shoot mass among the three alpine habitats (Fig. 2, Table 5). Seedlings grown in *Salix* soils were generally heavily colonized by ECMF and also tended to have high shoot biomass, with little difference between host^+^ and host^−^ soils. Seedlings grown in the *Arctostaphylos* soils tended to be intermediate in both level of ECMF colonization and shoot biomass and also lacked separation between host^+^ and host^−^ soils. However, seedlings grown in *Betula* soils ranged from relatively small and poorly colonized, to larger, well colonized seedlings. The former were most often those grown in host^−^ soils and the latter in host^+^ soils. Slopes of the regression lines from the different groupings of soil samples were significantly different (p = 0.040), indicating that the influence of ECM colonization on bioassay seedling growth varies among soil types. The greatest influence was seen in all host^−^ soils combined and the smallest influence was in *Arctostaphylos* soils (Table 5).

In the multiple regressions of bioassay seedling growth against ECMF and soil factors in the different soils, nitrate was generally the most important after ECMF colonization in explaining bioassay shoot mass (Table 6). The relative importance of nitrate ranged from negligible, as in the analysis of all alpine soil types and *Betula* habitat soils only, to nearly equivalent to percent ECMF colonization, as in the analysis of *Arctostaphylos* soils. pH was an important factor when all alpine soils were included, and the amount of soil phosphorous was important in the analyses of all alpine soils, all host^−^ soils, and *Birch* soils. The multiple regressions explained as much as 75.9% of the variation in bioassay seedling shoot mass when only *Betula* habitat soils were included, and as little as 34.4% when only *Arctostaphylos* habitat soils were analyzed.

**Table 6:** Standardized regression coefficients (Beta values) and p values for individual factors, and the adjusted R^2^ and overall p values for statistically significant multiple regression equations relating seedling shoot mass to independent variables for different groupings of soil samples.

Origin of bioassay soil	n^a^	ECM^b^		Nitrate		pH		Phosphorus		R^2^	Overall p
		Beta	p	Beta	p	Beta	p	Beta	p		
All habitats (host^+^ and host^−^)	89	0.728	**<0.000**			−0.154	**0.018**	0.200	**0.003**	0.645	**<0.000**
All habitats (host^+^ only)	45	0.775	**<0.000**					0.273	**0.011**	0.536	**<0.000**
All habitats (host^−^ only)	44	0.725	**<0.000**					0.187	**0.036**	0.707	**<0.000**
*Betula* habitat	45	0.853	**<0.000**					0.186	0.058	0.746	**<0.000**
*Arctostaphylos* habitat	45	0.449	**0.006**	0.403	**0.013**					0.344	**0.001**
*Salix* habitat	44	0.571	**0.001**	0.282	0.068					0.391	**0.001**

Shoot mass and ECM colonization data were log and arcsine transformed, respectively.

Statistically significant p values are in bold (α = 0.05).

a Number of soil samples included in each grouping.

b Percentage ectomycorrhizal colonization.

Bioassay root length was not correlated with any of the ECMF factors in the simple regressions. Although there appeared to be a weak negative correlation between root length and overall soil nutrient status, no significant multiple regression model was found to explain variation in root length within any combination of soil types. On average, bioassay seedling roots were longest in forest soils and shortest in *Arctostaphylos* soils. Within habitats, root length was always greater in host^−^ soils than in host^+^ soils and significantly so in the case of *Arctostaphylos* (Table 1).

Differences in soil and ECMF factors among sites (within habitats) were either non-significant, or showed no significant interactions between site and the measured variable.

## Discussion

We found that ECMF were readily available to black spruce above treeline and that important differences in colonization levels, richness, diversity and species composition occur both among habitats and among soils within habitats. Along with climate and soil fertility, the percentage of fine roots colonized by ECMF is an important factor in host plant establishment and growth, as higher colonization levels may allow greater nutrient transport to the host [58], [59]. However, it has long been assumed that mycorrhizal colonization will only lead to improved plant growth if the nutritional benefits outweigh the expenditure of carbon [60]. From this, it follows that high colonization rates are only advantageous when soil nutrient levels are low, and that colonization may impede plant growth when nutrients are more available [61]. Conversely, recent data analysis indicates that host plants generally have a surplus of carbon available for symbiosis with ECMF, and that high ECMF colonization levels are generally related to improved nutrient uptake and do not limit plant growth through carbon loss [62]. Accordingly, our results indicate that soil nutrients and ECMF colonization acted synergistically on bioassay seedling growth, resulting in the greatest growth in soils in which both nutrient and colonization levels were high.

Positive correlations between ECMF species richness and host plant growth have also been demonstrated in artificial systems [63], [64]. This effect is thought to be due to improved nutrient uptake, as fungal species differ in their abilities to obtain nutrients and supply them to their host [65]–[67]. Therefore, under certain conditions, colonization by more fungal species should result in more efficient access to soil resources, and increased plant growth. We did not find a correlation between the growth of our bioassay seedlings and ECMF species richness or diversity, likely because ECMF richness per seedling was not dramatically different across soils (generally one or two species). This factor may become more important as seedlings age in the field and begin to acquire a greater diversity of ECMF. We are aware that the richness and diversity of ECMF documented on our bioassay seedlings may not reflect the actual diversity of ECMF colonizing the shrubs on the site, as some of these may exhibit a level of host preference that would preclude them from colonizing black spruce seedlings. However, previous work on ECMF communities in the Canadian Rockies indicates that the alpine zone is dominated by generalists with the potential to colonize both woody alpine plants and conifer seedlings [27].

ECMF species composition can also be an important factor in seedling establishment, as the response of any given host plant to colonization by different fungal species can vary greatly [68], even when colonization levels are equivalent [69]. Further, as regenerating seedlings are thought to be well adapted to the ECMF species colonizing their parent trees [70], seedlings regenerating in alpine soils which support an ECMF community similar to those found in the mature forest may have an advantage over those in soils with ECMF dissimilar from the parent tree. In fact, assessment of ECMF species colonizing *Arctostaphylos uva-ursi* above treeline indicates little host specificity; a characteristic which may be related to the successful afforestation of sites dominated by *Arctostaphylos.* [22]).

Although we did not see an obvious relationship between species composition and bioassay seedling growth, we did find that the ECMF communities colonizing our bioassay seedlings grown in all host^−^ soils (and in *Salix* host^+^ soils) were characterized by high levels of *Laccaria* and *Thelephora.* These are common pioneer fungi, with broad host ranges, high germination rates and the ability to quickly form ECM [71]–[73]. These two fungi therefore appear to be important components of the alpine ECMF spore bank, able to efficiently colonize the roots of establishing plants.

Our soil bioassay approach allowed us to determine the influence of ectomycorrhizal colonization on the growth of spruce seedlings. We found colonization levels to be strongly correlated with bioassay seedling growth, explaining over half of the variation in shoot mass data when all alpine soil types were analyzed together. This effect varied among habitats however, and in subsets of soil types including only *Salix* or only *Arctostaphylos* soils, colonization levels explained relatively small amounts of differences in seedling growth. Although we assume that much of the remaining variation was due to differences in soil nutrients, the inclusion of soil nutrient data did little to improve the regression models for these two habitats. This may be due to differences in soil temperature and moisture between the host^+^ and host^−^ soils, which may have resulted in different nitrification rates and therefore differences in the major form of nitrogen (ammonium as opposed to nitrate) available to seedlings. However, in *Betula* soils ECMF colonization alone explained a large proportion of the variation in seedlings growth, likely because nitrogen was fairly homogenous throughout the habitat (although phosphorus varied).

The possibility does exist that the observed correlation between ECMF colonization levels and bioassay seedling shoot mass was partly due to soil nutrient levels, in that higher nutrient levels would result in larger seedlings with more carbon available to ECMF, resulting in higher colonization levels. However, soil nutrient levels and ECM colonization were not correlated.

With respect to the influence of ectomycorrhizal shrubs on the availability of ECMF above treeline, we found that seedlings grown in all of the soil types (except the glacial moraine soils) became fairly well colonized by ECMF, but colonization was generally higher in host^+^ soils than in host^−^ soils within a habitat (although only significantly so for *Betula* soils). The higher colonization levels in host^+^ soils is likely due to the greater inoculum potential provided by a combination of ectomycorrhizal networks and spores in host^+^ soils, as opposed to only spore inoculum in host^−^ soils, where ectomycorrhizal networks could not extend [30]. Nevertheless, colonization levels of seedlings grown in some host^−^ soils could be surprisingly high, even though they supported only a few dominant ECMF species.

Colonization levels of seedlings grown in host^−^ soils were positively correlated with those of corresponding host^+^ soils within the same habitat. For example, *Salix* soils had the highest inoculum potential of any host^+^ soil and the highest of any host^−^ soil, while *Arctostaphylos* soils had the lowest inoculum potential in both host^+^ and host^−^ soils. Also, although dominated by *Laccaria* and *Thelephora*, species composition in host^−^ soils was generally a subset of that found in host^+^ soils from the same habitat. These observations imply that inoculum potentials of spore banks in the host^−^ soils may be influenced by the presence of nearby host^+^ soils, where shrubs supply fixed carbon to ectomycorrhizal networks for the production of fruiting bodies [74] and therefore spores, which are then distributed locally.

Functional ECMF spore banks (including *Laccaria* spores) were detected within 40 years of glacial retreat [75] and ECMF spores can act as an inoculum source for several years [37]. In fact, spores of many ECMF species may remain dormant until germination is triggered by the presence of a suitable host plant, such as an establishing seedling [73], [76], [77]. This indirect influence of ectomycorrhizal shrubs on soils not vegetated by ectomycorrhizal hosts appears to occur at the scale of our sampling sites, but does not seem to extend further, such as into the glacial moraine. This is in agreement with studies indicating that the vast majority of wind dispersed ECMF spores travel only one meter from their fruiting bodies [31] and that ECM colonization can decrease dramatically within 20 m of a forest edge [18]. However, a recent spore trapping study has demonstrated high interspecific variation in both ECMF spore production and dispersal, with the spores of some species colonizing seedlings 1 km or more from their source [78].

Shrub density is currently increasing in many high elevation and latitude locations [79]. This should mean a concomitant increase in the range of associated ectomycorrhizal networks and the fruiting bodies and wind dispersed spores they produce. Therefore, as temperatures increase, spores of ectomycorrhizal fungi may represent the leading edge of the migration of woody vegetation (both shrubs and trees) into alpine and tundra habitats.

## Supporting Information

Table S1 **Soil nutrients, pH levels, organic matter contents (OM) and cation exchange capacities (CEC) for each soil sample.**
(DOC)

Table S2 **Proportions of ECM morphotypes colonizing bioassay seedlings grown in soils from each habitat.**
(DOC)
