# Supplementary material for: Availability of Ectomycorrhizal Fungi to Black Spruce above the Present Treeline in Eastern Labrador
Source: PLoS One. 2013 Oct 29;8(10):e77527. doi: 10.1371/journal.pone.0077527 (PMC3812278; doi:10.1371/journal.pone.0077527)
Supplement: Table S1 — Soil nutrients, pH levels, organic matter contents (OM) and cation exchange capacities (CEC) for each soil sample. (DOC) [file pone.0077527.s001.doc]

**Table S1**. Soil nutrients, pH levels, organic matter contents (OM) and cation exchange capacities (CEC) for each soil sample.

|  | **pH** | **OM** | **CEC** | **P_2_O_5_** | **NO_3_** | **N** | **K_2_O** | **Ca** | **Mg** | **Na** | **S** | **Al** | **Fe** | **Mn** | **Cu** | **Zn** | **B** |
| --- | --- | --- | --- | --- | --- | --- | --- | --- | --- | --- | --- | --- | --- | --- | --- | --- | --- |
| **SA1-S3** | **4.6** | **17.20** | **11.67** | **70.00** | **9.00** | **0.86** | **86.83** | **113.33** | **34.67** | **16.83** | **42.83** | **1811.02** | **363.67** | **5.00** | **1.99** | **2.23** | **0.19** |
| **SA2-S3** | **4.4** | **22.40** | **12.00** | **63.67** | **26.90** | **1.17** | **92.50** | **152.33** | **43.67** | **22.00** | **29.83** | **1386.69** | **405.67** | **5.00** | **1.26** | **2.43** | **0.18** |
| **SA3-S3** | **4.8** | **10.40** | **9.00** | **51.67** | **19.40** | **0.53** | **74.50** | **49.33** | **19.17** | **19.67** | **40.17** | **2224.57** | **221.00** | **2.00** | **1.33** | **1.37** | **0.11** |
| **SA4-S3** | **4.5** | **14.37** | **10.00** | **58.33** | **21.33** | **0.71** | **74.50** | **67.83** | **20.67** | **21.67** | **37.17** | **1771.61** | **229.67** | **1.33** | **0.83** | **1.30** | **0.12** |
| **SA5-S3** | **4.7** | **10.93** | **9.67** | **101.17** | **17.60** | **0.52** | **98.00** | **61.00** | **18.17** | **27.33** | **52.17** | **2122.26** | **249.67** | **2.33** | **0.99** | **1.37** | **0.16** |
| **SA1+S3** | **4.7** | **25.13** | **11.67** | **50.83** | **1.67** | **1.12** | **166.83** | **187.17** | **66.83** | **18.67** | **21.17** | **1042.64** | **341.00** | **5.33** | **1.11** | **4.53** | **0.20** |
| **SA2+S3** | **4.6** | **29.30** | **12.67** | **50.33** | **3.93** | **1.19** | **234.17** | **303.00** | **111.17** | **18.17** | **22.83** | **921.43** | **316.67** | **9.33** | **1.10** | **7.70** | **0.21** |
| **SA3+S3** | **5** | **12.00** | **10.33** | **54.00** | **0.93** | **0.61** | **138.00** | **175.83** | **48.00** | **23.00** | **34.33** | **1744.86** | **267.00** | **3.67** | **0.96** | **3.20** | **0.16** |
| **SA4+S3** | **4.8** | **28.37** | **11.00** | **63.17** | **2.30** | **1.20** | **167.33** | **192.33** | **53.83** | **18.83** | **24.33** | **1230.43** | **228.00** | **4.33** | **0.64** | **3.50** | **0.15** |
| **SA5+S3** | **4.8** | **14.33** | **10.33** | **62.17** | **1.87** | **0.65** | **93.83** | **78.17** | **23.33** | **19.17** | **42.17** | **2188.44** | **283.67** | **2.00** | **0.71** | **1.93** | **0.12** |
| **AR1-S3** | **4.8** | **12.17** | **8.33** | **37.67** | **5.73** | **0.52** | **65.67** | **79.33** | **16.33** | **37.83** | **40.83** | **2134.38** | **186.33** | **2.00** | **0.61** | **1.43** | **0.15** |
| **AR2-S3** | **4.9** | **11.77** | **7.33** | **28.00** | **4.20** | **0.48** | **29.17** | **62.17** | **11.17** | **10.50** | **28.17** | **1983.86** | **201.33** | **1.33** | **0.47** | **0.77** | **0.16** |
| **AR3-S3** | **4.5** | **13.37** | **7.33** | **44.50** | **9.47** | **0.60** | **52.67** | **95.33** | **18.33** | **12.67** | **34.00** | **1921.55** | **214.00** | **2.33** | **0.40** | **1.73** | **0.16** |
| **AR4-S3** | **4.6** | **11.17** | **6.67** | **29.50** | **7.10** | **0.39** | **32.17** | **44.00** | **10.83** | **9.00** | **34.50** | **1938.29** | **158.00** | **1.33** | **0.51** | **0.87** | **0.10** |
| **AR5-S3** | **4.6** | **8.20** | **6.33** | **25.83** | **9.13** | **0.48** | **26.00** | **17.50** | **7.50** | **10.17** | **53.50** | **2213.38** | **185.00** | **1.00** | **0.46** | **0.33** | **0.13** |
| **AR1+S3** | **4.9** | **12.60** | **10.00** | **27.50** | **2.93** | **0.66** | **64.33** | **154.50** | **17.83** | **17.67** | **33.83** | **2303.02** | **174.33** | **3.67** | **0.68** | **3.97** | **0.14** |
| **AR2+S3** | **5.2** | **11.07** | **8.67** | **40.83** | **1.57** | **0.60** | **48.67** | **120.17** | **16.67** | **8.67** | **29.33** | **1989.25** | **173.33** | **2.00** | **0.46** | **2.37** | **0.13** |
| **AR3+S3** | **4.6** | **28.53** | **12.00** | **75.00** | **0.57** | **1.37** | **72.67** | **369.33** | **27.50** | **9.00** | **19.00** | **992.66** | **134.67** | **1.33** | **0.20** | **7.07** | **0.08** |
| **AR4+S3** | **4.8** | **24.90** | **11.00** | **44.17** | **0.97** | **1.02** | **65.83** | **313.50** | **32.00** | **10.50** | **22.83** | **1315.98** | **193.00** | **2.00** | **0.38** | **7.03** | **0.12** |
| **AR5+S3** | **5.1** | **11.57** | **8.33** | **30.50** | **1.10** | **0.49** | **41.17** | **155.67** | **18.00** | **9.83** | **39.83** | **1978.32** | **242.00** | **2.00** | **0.33** | **2.77** | **0.11** |
| **SB1-S3** | **4.7** | **15.60** | **7.33** | **43.50** | **4.87** | **0.75** | **45.50** | **36.50** | **16.33** | **12.00** | **30.50** | **1871.84** | **208.00** | **1.00** | **0.31** | **0.80** | **0.15** |
| **SB2-S3** | **4.7** | **18.17** | **7.33** | **54.33** | **9.73** | **0.83** | **84.33** | **50.83** | **19.50** | **14.50** | **33.83** | **1728.09** | **206.67** | **1.33** | **0.42** | **0.93** | **0.09** |
| **SB3-S3** | **4.7** | **20.60** | **9.33** | **56.67** | **3.53** | **0.95** | **60.00** | **37.33** | **16.50** | **10.33** | **40.00** | **2018.00** | **222.33** | **1.00** | **0.27** | **0.67** | **0.14** |
| **SB4-S3** | **4.6** | **23.03** | **9.33** | **38.83** | **5.17** | **1.05** | **54.83** | **82.50** | **21.67** | **12.67** | **22.83** | **1617.43** | **297.00** | **1.33** | **0.32** | **0.97** | **0.13** |
| **SB5-S3** | **4.9** | **18.83** | **7.67** | **39.00** | **5.27** | **0.97** | **66.33** | **36.50** | **17.83** | **15.83** | **30.83** | **1863.56** | **159.33** | **0.67** | **0.25** | **1.13** | **0.10** |
| **SB1+S3** | **4.9** | **37.70** | **11.33** | **136.50** | **1.13** | **1.80** | **150.17** | **381.67** | **54.00** | **23.83** | **22.50** | **902.71** | **153.33** | **2.67** | **0.28** | **2.40** | **0.10** |
| **SB2+S3** | **4.5** | **29.73** | **11.33** | **77.67** | **2.83** | **1.51** | **108.33** | **200.83** | **34.17** | **15.50** | **24.17** | **1380.26** | **200.33** | **1.33** | **0.32** | **2.00** | **0.09** |
| **SB3+S3** | **4.3** | **40.20** | **10.67** | **54.17** | **1.40** | **1.47** | **104.17** | **290.33** | **57.33** | **13.83** | **9.67** | **506.36** | **112.00** | **2.00** | **0.34** | **4.23** | **0.09** |
| **SB4+S3** | **4.5** | **32.80** | **9.00** | **56.33** | **1.57** | **1.41** | **77.50** | **120.67** | **29.17** | **17.00** | **17.33** | **1011.41** | **202.00** | **1.33** | **0.24** | **1.03** | **0.07** |
| **SB5+S3** | **4.8** | **19.97** | **9.00** | **35.67** | **6.37** | **0.95** | **65.50** | **93.50** | **31.00** | **11.83** | **24.83** | **1642.67** | **261.67** | **2.00** | **0.33** | **2.20** | **0.11** |
| **FO1+S3** | **4.4** | **44.50** | **9.00** | **33.67** | **1.67** | **1.32** | **95.00** | **255.67** | **41.83** | **15.33** | **11.33** | **853.93** | **181.33** | **2.00** | **0.19** | **2.37** | **0.13** |
| **FO2+S3** | **4.2** | **35.23** | **11.00** | **32.33** | **1.03** | **0.87** | **73.17** | **227.00** | **54.33** | **17.33** | **10.83** | **749.65** | **155.67** | **1.67** | **0.16** | **1.93** | **0.12** |
| **FO3+S3** | **4.1** | **25.30** | **11.00** | **57.17** | **0.50** | **0.75** | **100.67** | **281.00** | **65.83** | **21.00** | **11.33** | **619.81** | **123.33** | **3.00** | **0.28** | **1.60** | **0.05** |
| **FO4+S3** | **4.4** | **17.77** | **9.67** | **43.50** | **0.20** | **0.62** | **96.67** | **204.83** | **48.17** | **29.33** | **18.50** | **1251.25** | **322.00** | **5.00** | **0.32** | **1.30** | **0.14** |
| **FO5+S3** | **4.4** | **12.77** | **8.33** | **49.00** | **0.20** | **0.43** | **61.83** | **200.50** | **43.17** | **17.00** | **14.67** | **1043.41** | **235.33** | **1.67** | **0.19** | **1.70** | **0.08** |

All values in ppm except for pH, OM and N (percent) and CEC (meq/100mg)

**S**, *Salix*; **B**, *Betula*; **A**, *Arctostaphylos*; **F**, Forest; **+**, host present; -, host absent
