# Supplementary material for: Availability of Ectomycorrhizal Fungi to Black Spruce above the Present Treeline in Eastern Labrador
Source: PLoS One. 2013 Oct 29;8(10):e77527. doi: 10.1371/journal.pone.0077527 (PMC3812278; doi:10.1371/journal.pone.0077527)
Supplement: Table S2 — Proportions of ECM morphotypes colonizing bioassay seedlings grown in soils from each habitat. (DOC) [file pone.0077527.s002.doc]

**Table S2.** Proportions of ECM morphotypes colonizing bioassay seedlings grown in soils from each habitat.

| **Morphotype** | **Forest** | **Betula** | | **Arctostaphylos** | | **Salix** | |
| --- | --- | --- | --- | --- | --- | --- | --- |
|  |  | **Host^+^** | **Host**^−^ | **Host^+^** | **Host**^−^ | **Host^+^** | **Host**^−^ |
| *Cenococcum* | 0.021 | 0.040 | 0.011 | 0.041 | 0.010 | 0.120 | 0.012 |
| *Elaphomyces* | 0.123 | 0.070 | 0 | 0 | 0 | 0 | 0 |
| *Hydnotrya* | 0.003 | 0 | 0 | 0 | 0 | 0 | 0 |
| *Inocybe* | 0.000 | 0 | 0 | 0.0340 | 0.035 | 0 | 0 |
| *Inocybe*-like | 0.023 | 0 | 0 | 0 | 0 | 0 | 0 |
| *Laccaria*/*Thelephora* | 0.054 | 0.431 | 0.846 | 0.072 | 0.828 | 0.757 | 0.964 |
| *Lactarius* | 0.057 | 0.131 | 0 | 0 | 0 | 0 | 0 |
| *Meliniomyces* | 0.017 | 0 | 0.023 | 0 | 0 | 0 | 0 |
| *Peziza* | 0 | 0 | 0 | 0 | 0 | 0.078 | 0 |
| *Pseudotomentella* | 0.003 | 0 | 0 | 0 | 0 | 0 | 0 |
| *Sebacina* | 0.195 | 0.324 | 0.020 | 0.444 | 0.088 | 0.033 | 0.021 |
| *Tomentella* | 0 | 0.003 | 0 | 0.028 | 0.005 | 0.012 | 0.003 |
| *Tomentellopsis* | 0 | 0 | 0 | 0.024 | 0 | 0 | 0 |
| *Trichophea* | 0.226 | 0 | 0 | 0 | 0 | 0 | 0 |
| *Tylospora* | 0.276 | 0 | 0 | 0.350 | 0 | 0 | 0 |
